# Effect of Barley Antifreeze Protein on Dough and Bread during Freezing and Freeze-Thaw Cycles

**DOI:** 10.3390/foods9111698

**Published:** 2020-11-19

**Authors:** Xiangli Ding, Tingting Li, Hui Zhang, Chengran Guan, Jianya Qian, Xiaoyan Zhou

**Affiliations:** 1 School of Food Science and Engineering & School of Tourism and Culinary Science, Yangzhou University, Yangzhou 225127, China; dingxl@yzu.edu.cn (X.D.); 004072@yzu.edu.cn (T.L.); crguan@yzu.edu.cn (C.G.); jyqian@yzu.edu.cn (J.Q.); MZ120191369@yzu.edu.cn (X.Z.); 2 State key Laboratory of Food Science and Technology & School of Food Science and Technology, Jiangnan University, Wuxi 224122, China

**Keywords:** barley, antifreeze protein, dough, freezing, freeze-thaw

## Abstract

In order to verify the cryoprotective effect of an antifreeze protein (BaAFP-1) obtained from barley on bread dough, the effect of BaAFP-1 on the rheological properties, microstructure, fermentation, and baking performance including the proofing time and the specific volume of bread dough and bread crumb properties during freezing treatment and freeze-thaw cycles were analysed. BaAFP-1 reduced the rate of decrease in storage modulus and loss modulus values during freezing treatment and freeze-thaw cycles. It influenced the formation and the shape of ice formed during freezing and inhibited ice recrystallization during freeze-thaw. BaAFP-1 maintained gas production ability and gas retention properties, protected gluten network and the yeast cells from deterioration caused by ice formation and ice crystals recrystallisation in dough samples during freezing treatment and freeze-thaw treatment. It slow down the increase rate of hardness of bread crumb. The average area of pores in bread crumbs decreased significantly (*p* < 0.05) as the total number of pores increased (*p* < 0.05), and the addition of BaAFP-1 inhibited this deterioration. These results confirmed the cryoprotective activity of BaAFP-1 in bread dough during freezing treatment and freeze-thaw cycles.

## 1. Introduction

Frozen dough technology used in the baking industry can both supply oven-fresh bakery products and also improve labour conditions, thus gained extensive attention. However, both the freezing process and frozen storage decrease dough quality. The formation of ice crystals during freezing treatment and ice recrystallisation during frozen storage, due to low and fluctuating temperatures, result in the deterioration of hydrated gluten integrity [1,2], the alteration of the structural and functional properties of wheat starch [3], and affect the viability and activity of yeast [4]. Various Problems have arise during the production of bread made from frozen dough, including the gradual loss of dough strength, decreased CO_2_ retention capacity, reduced yeast activity. Reflect on final products properties, a longer fermentation time, decreased loaf volume, and a deterioration in the bread crumb texture can be observed [5,6,7]. 

Various food additives, including emulsifiers, enzymes, hydrocolloids, and antifreeze proteins (AFPs), have been used with the aim of improving the rheological and structural properties and baking performance of frozen dough [8,9,10]. Among these additives, AFPs are novel food ingredients that have attracted much interest owing to their effects on the properties of frozen dough [11,12,13]. AFPs, alternatively ice-structuring proteins (ISPs) or thermal hysteresis proteins, are a family of proteins, which could lower the freezing point of poikilothermic organisms in a non-equilibrium manner, thus protect them from freezing [14,15]. This is referred to as thermal hysteresis activity (THA). THA is only one of the effects mediated by these proteins, and it may not be the most common effect. AFPs also alter the way ice crystals form, which is referred to as ice crystal morphology modification activity [16]. Moreover, they inhibit ice recrystallisation by incorporating with the ice due to their affinity for the ice crystal surface, named as ice recrystallisation inhibition (IRI) [17]. These unique properties of AFPs make them excellent candidate for natural ice modulators in food cryopreservation. 

AFPs have been found in fish, bacteria, and plants, et al. [18]. The diverse structures and compositions of AFPs result in large differences in their activities and functional mechanisms. Plant AFPs generally have relatively low THA (0.2–0.4 °C) compared with AFPs from fish (0.7–1.5 °C) and insects (3–6 °C). However, they show extraordinarily high IRI activity [17], with a 100–500 times lower concentration of AFPs required for their IRI effect than for freezing point depression. Therefore, these AFPs are cost-effective if they were used as food ingredients [15,19]. It has been proposed that plant AFPs function through IRI rather than by its THA [20]. This characteristic makes them ideal candidates for the cryopreservation of oocytes, embryos, and frozen food, in which ice recrystallisation has the greatest negative effect on preservation. Although plant AFPs are widely found in winter and spring rye, winter and spring wheat, and winter and spring canola, et al., they have only been purified from carrot, peach tree, winter rye, winter wheat, oats, bittersweet night shade, *L. perenne*, *A. mongolicus*, and sea buckthorn. However, few studies on their food preservation effects have been published. The incorporation of plant AFPs from winter wheat and carrots has been shown effective in influencing the gassing rate, the total amount of gas produced in frozen dough, maintaining loaf volume, improving the texture properties of bread during frozen storage; decreasing the rate of structural deterioration; and retarding the decrease of freeze-thaw stability in terms of syneresis and the hardness of corn and wheat starch gels. These results demonstrate that the application of plant AFPs as food preservatives can improve the quality of frozen foods during process, storage, and transport. Therefore, studies on the effects of plant AFPs during cryopreservation, aimed at revealing the structure/function relationship of plant AFPs, will be of great interest. 

A barley antifreeze protein (BaAFP-1) was extracted and purified from barley (*Hordeum vulgare L.*) [21]. Previous study showed that it could decrease dough deterioration by influencing its freezing-thawing parameters, including freezing and melting temperatures and enthalpy, freezable water content, and glass transition temperature. BaAFP-1 could also influence water mobility and water distribution in frozen dough during freezing process and freeze-thaw cycles [18]. To verify the effect of BaAFP-1 on the end-use properties of dough, the effect of BaAFP-1 on dough samples during freezing process and freeze-thaw treatment was determined. These data will add to the existing information on the cryoprotective activity of AFPs, providing further insight into the action mechanism of plant AFPs.

## 2. Materials and Methods 

### 2.1. Materials 

BaAFP-1 was extracted from barley (*Hordeum vulgare L.*) by infiltration-centrifugation with phosphate buffer (pH 7.2, 0.15 M NaCl, 1:5 (*w*/*v*) for 10h. Then the pooled centrifugal fluid and the infiltrating buffer was concentrated and desalted by ultra-filtration (Cole-Parmer Master Flex L/S Digital Drive, Cole-Parmer Instrument. Vernon Hills, IL, USA). Purification was conducted with ammonium sulphate precipitation (50–100% cut), ion-exchange (ANS-Seharose Fast-Flow column), gel filtration (Hiload Sephadex G-75 gel filtration column) and hydrophobic chromatography (Phenyl Sepharose High Performance column). BaAFP-1 obtained was pooled, lyophilized for use. Flour, sugar, salt and butter were purchased from the local market.

### 2.2. Dough Preparation and Storage

Dough samples were prepared use the method described before [18]. Formulation of control dough was comprised of 100 g wheat flour, 4 g sugar, 1.5 g salt, 60 g water, and 4 g butter. AFPs was added in BaAFP-1 dough at the ratio of 0.5% (flour basis). All ingredients except butter were mixed at the same time, then butter was added when gluten in the dough was partly formed. When gluten in the dough was fully extended, the dough was rested, divided, shaped and wrapped in a polyethylene sheet. They were called control dough samples and BaAFP-1 dough samples, respectively. The frozen dough samples were immediately frozen at −30 °C for 2 h, then stored at −18 °C. Freeze-thaw cycle, consisted of partially thawing the frozen dough samples at room temperature until the centre temperature was 15 °C, then subjected to frozen storage again at −18 °C for 24 h, were conduct used the method described before to mimic the temperature fluctuations [18]. Samples were named Fresh, C1, C2, C3, and C4, respectively, in which Fresh refers to fresh dough samples, and in C1, C2, C3 and C4, represents dough samples endured 1 to 4 freeze-thaw times. No yeast was added to dough samples used for dynamic rheological measurement and microstructure measurement in order to eliminate the influence of fermentation during measurement.

### 2.3. Dynamic Rheological Measurement of Dough Samples

Dough dynamic rheological property was measured using a AR-G2 rheometer (TA Instruments, New Castle, DE, USA) using the slightly modified methods of Li and Ribotta [8,9]. Parallel plate geometry of 20 mm diameter and 1 mm gap was employed. Samples were placed on the lower plate, and its rim was coated with Vaseline. Before starting the measurement, samples were rest 5 min at 25 °C, to relax normal stresses induced during sample loading. Frequency sweep tests were performed over the frequency range of 0.01–40.00 Hz, 0.5% deformation. The storage modulus (G’), loss modulus (G”) and tan *δ* (G”/G’) were recorded. 

### 2.4. Microstructure Measurement of Dough Samples 

Microstructure observations were performed on frozen dough samples with and without addition of BaAFP-1 by SEM (model SCD005, Bal-Tec, Liechtenstein). Frozen dough samples were frozen dried use a freeze dry system (−80 °C, <10 Pa, Labconco, Kansas City, MO, USA) prior to SEM analysis. Then fractured and sputter coated with gold-palladium alloy and their surface structures were viewed at an accelerating voltage of 5kV. Representative micrographs was taken at magnification of ×300 and ×1200, respectively. 

### 2.5. Fermentation Properties Determination of Dough Samples

Fermentation properties of dough samples were measured with a rheofermentometer (F3, Chopin, Villeneuve-La-Garenne, France). The test was conducted on a using the slightly modified methods of Roth [22]. 150 g dough samples were put into a fermentation basket, then loaded a 1.5 kg cylindrical piston and then sealed the proofing chamber hermetically. Fermentation properties of dough samples were analyzed at 37 °C for 3 h. Development of the dough and gaseous release curves were recorded. Maximum dough height (Hm), time to reach maximum dough height (T_1_), maximum height of gaseous release (H’m), total volume (V(CO_2_)), and retention coefficient (R) were determined.

### 2.6. Baking Properties Determination of Dough Samples

Baking properties including the proofing time of dough and the specific volume of baked bread was determined, respectively. Paned dough samples were placed into a fermentation cabinet. Proofing time required at a fermentation condition of 38 °C and 85% RH was recorded with a fixed proof height (~1 cm below the pan sidewall). The loaves were baked at 195 °C/185 °C for 13 min after they reached their appropriate proofing time, respectively. They were cooled for 1 h prior to further testing. Then the weight and loaf volume of each bread loaf was determined by millets displacement. The specific volume (mL/g) of baked bread were calculated as quotient of loaf volume (mL) and weight (g) of each bread sample.

### 2.7. Hardness Analysis of Bread Crumb

Hardness analysis of bread crumb was conducted using the method of Kim with slight modifications [10]. A two cycle bread crumb compression text were performed on two bread slices (1-cm thick) using a TA-XT2i texture analyzer (Stable Micro System, Surrey, UK) equipped with a P25 Aluminum Platen probe of 2.5 cm diameter. The test was conducted under the following conditions: Pre-test speed 1 mm/s, test speed 0.8 mm/s, post-test speed 0.8 mm/s, trigger type auto 5 g, and 50% compression. The first compression cycle was recorded to be hardness. 

### 2.8. Crumb Grain Features Observation of Bread

Bread slices (1-cm thick) were scanned using a flatbed scanner (CanoScan LiDE25, Canon, Lake Success, NY, USA) at a resolution of 600 dpi. A field of view 165 × 165 was cropped from the crumb grain feature image using Adobe Photoshop CS5. The cropped images were converted from RGB mode into gray scale, converted into binary image and then analyzed with MATLAB 2007b software program (Program S1) using the slightly modified image analysis method used by Gao [23]. Distribution of bread pore was quantified and recorded.

### 2.9. Statistical Analysis 

Data were expressed as Mean ± SD. The Origin software was used in the data treating. Statistical analyses were done by one-way analysis of variance (ANOVA) with Duncan post hoc test using SPSS for Windows, version 16.0 (SPSS Inc. 1999), and *p* < 0.05 was chose as significance level.

## 3. Results and Discussion

### 3.1. Effect of BaAFP-1 on the Dynamic Rheological Properties of Dough Samples

**Figure 1:**
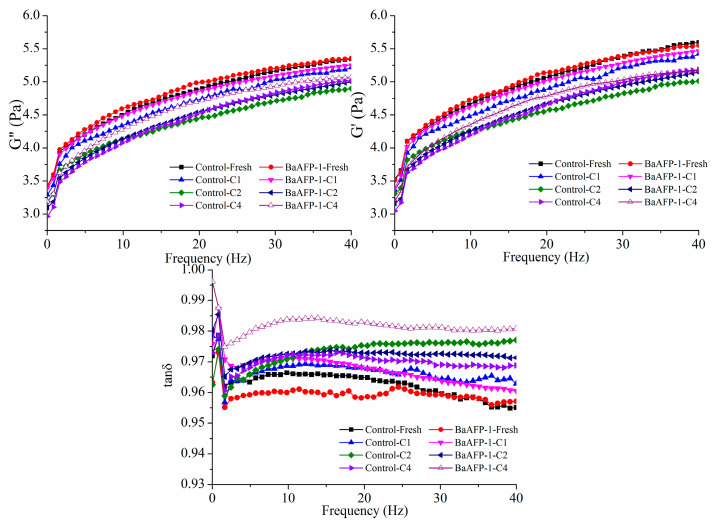
Effect of BaAFP-1 on the dynamic rheological of dough samples.

Due to its characteristic and sensitive response in structural variations, dynamic rheological testing has become a preferred approach in the structure and fundamental properties study of dough samples [24]. Dynamic oscillation is a general test in the rheological properties determination of dough [25], because the G′ and G″ values show significant positive correlations with bread loaf volume [26]. It is generally considered that gliadins confer viscous properties [24]. In particular, the G′ value of gluten dough samples was highly related to bread-making performance, accounting for 73% of the variation in loaf volume. Meanwhile, glutenins was related to strength and elasticity, which are essential for the gases retaining property of bread dough [27,28]. The dynamic rheological properties of fresh, frozen, and freeze-thawed dough samples, with and without the addition of BaAFP-1, are shown in Figure 1. All dough samples showed a gel-type material model, with tan δ less than 1 for the entire frequency range. Both G′ and G″ values of all dough samples decreased, whereas tan δ increased after freeze-thaw treatment. Notably, the rate of decrease in G′ and G″ values was lower after the addition of BaAFP-1, as the G′ and G″ values of BaAFP-1-C4 dough samples were clearly higher than those of the control samples. Reduced G′ and G″ values during the freezing process have also been reported by Ribotta [9] and Jia [13]. Tan *δ* was lower in fresh, frozen, and BaAFP-1-C2 samples than in control samples, whilst a marked increase and decrease were observed in BaAFP-1-C4 and Control-C4 samples, respectively. Different trends in tan *δ* have previously been observed in dough samples after the addition of thermostable ISPs (TSISPs) [13]. This may be due to different activities and ice-binding surfaces of AFPs purified from different sources, resulting in different effects on G′ and G″ values. The decrease in G′ and G″ values of all dough samples after freezing and freeze-thaw treatment seems to be a comprehensive reflection of the change in the structure of proteins and the depolymerisation of glutenin aggregates caused by ice [6]. Previous study shown that BaAFP-1 has been shown to not only influence the freezable water content and water mobility, but also influence water distribution in dough samples [18]. Consequently, changes in the structure of proteins caused by ice formation and ice recrystallisation may be weakened by the addition of BaAFP-1. That may explain why the G′ and G″ values of all BaAFP-1-containing dough samples were higher than those of the control samples during freezing and freeze-thaw cycles. It has generally been shown that the tan δ values of dough samples made from high quality flour are lower than those of dough samples made from low quality flour [24]. It can be speculated that the existence of BaAFP-1 would improve the quality of the dough to some extent. Considering the role of glutenin and gliadin in the formation of gluten and the general tendency for changes in tan *δ*, which is calculated by dividing G″ by G′, the increase in tan *δ* indicates that G′ values decreased greater than G″ values. This suggests that the destructive effect of freezing and freeze-thaw treatment on glutenins was greater than that on gliadins. This is consistent with the conclusions reported by Wang [29]. The balance of dough visco-elasticity is the most important factor in high-quality bread making [30]. Changes in tan δ may also reflect the deterioration of dough properties. 

### 3.2. Effect of BaAFP-1 on the Microstructure of Dough Samples

**Figure 2:**
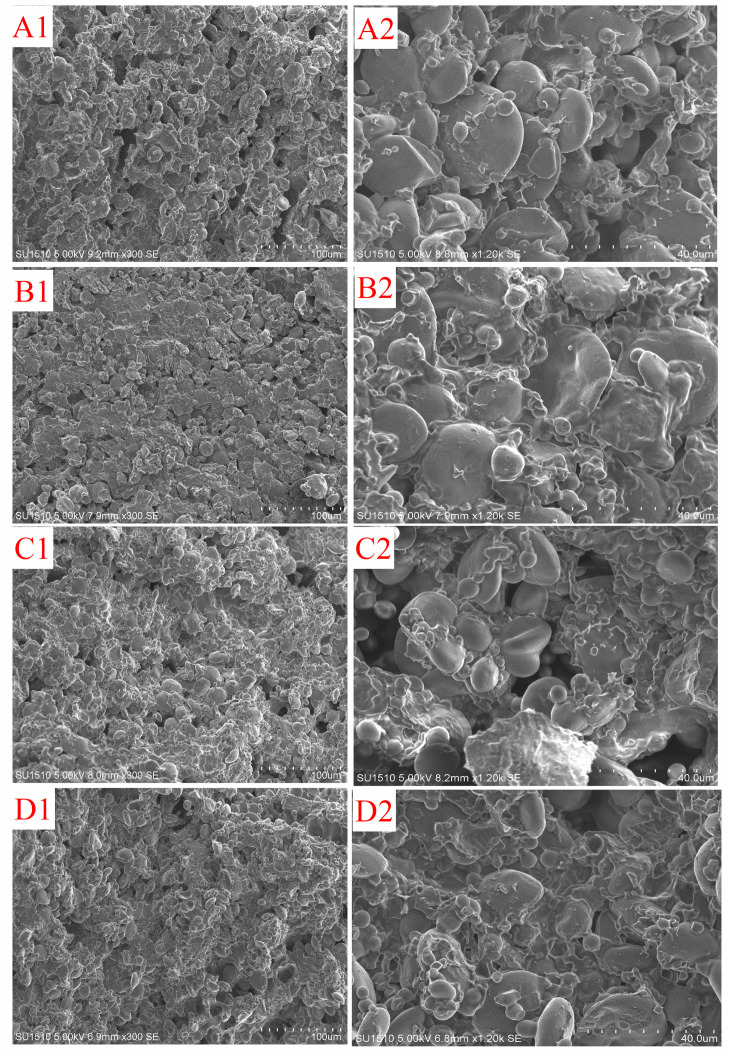
Effect of BaAFP-1 on the microstructure of dough samples. (**A**,**B**) stand for control and BaAFP-1 dough samples after frozen treatment, respectively; (**C**,**D**) stand for control and BaAFP-1 dough samples after freeze-thaw treatment, respectively; number 1 and 2 after (**A**–**D**) in the figure represent 300× and 1200× magnification, respectively.

The microstructure of dough samples after freezing process and freeze-thaw cycles was examined by SEM (Figure 2). The dominant feature was starch granules scattered within the gluten matrix. Voids formed by sublimation can be used to reflect the distribution of the ice formed in dough samples before freeze-drying. Some large voids were present in control dough samples (Figure 2(A1,A2)) means that the angular voids in it were less uniform. However, when BaAFP-1 was added to the dough, the gluten network was more continuous, with starch granules glued tightly to it (Figure 2(B1,B2)). Smaller voids were observed, and fewer large voids were visible. This indicated that BaAFP-1 influenced both the formation and the shape of the ice formed in dough samples during freezing, consequently strengthening the gluten network and decreasing the deterioration of the dough structure caused by ice crystal formation. After freeze-thaw cycles, more voids can be observed in control dough, and their size and shape became more irregular (Figure 2(C1,C2)). The detachment of starch granules from gluten was more severe, and starch granules seemed almost floating in the gluten. Moreover, few gluten fragments formed by gluten fracture were observed. Ice recrystallisation and gluten network cryoshrinkage occur in frozen food during temperature fluctuations, and these are the major factors that result in dough structure disruption [31]. Of these, ice recrystallisation is the most fatal hazard to the quality of frozen foods, as it can decrease the interstitial regions of the protein that separate adjacent ice crystals, leading to mechanical damage of the microstructure [9]. After freeze-thaw cycles, fewer voids and detached starch granules were observed in BaAFP-1-containing dough samples (Figure 2(D1,D2)) than in the control samples. This indicated that the presence of BaAFP-1 could inhibit ice recrystallisation during freeze-thaw. As a member of the AFP family, BaAFP-1 possesses the characteristic activities of AFPs, including thermal hysteresis, ice crystal morphology modification and ice recrystallisation inhibition, thus ice crystals can keep stable over a defined temperature range [32]. Plant AFPs have lower hysteresis activity than insect AFPs. They likely function by influencing ice crystal formation, modifying ice crystal shape, and inhibiting ice recrystallization [33]. Ice crystal formation and recrystallisation during freezing process and freeze-thaw cycles both lead to the gluten matrix damage and starch granules detachment. Therefore, the gas-holding ability and supporting capacity of the gluten framework deteriorates, dough proofing time prolongs, and the volume decreases during cooling after baking. The presence of BaAFP-1 may inhibit the deterioration effects of freezing and freeze-thaw cycles.

### 3.3. Effect of BaAFP-1 on the Fermentation Properties of Dough Samples

The rheofermentometer is a standard measuring instrument in studying flour behaviour during fermentation. Because fermentation is actually performed during the measurement, it is the only instrument that can give results closest to the real situation. F3 rheofermentometer was used to examine the fermentation properties of dough sample, and the results are summarised in Table 1. The recorded characteristics can be separated into three categories, as they reflect the dough properties from different angles. CO_2_ production (V(CO_2_) [mL]) indicates the gas production ability of dough samples, the maximum height of gaseous release (Hm′ [mm]) and R refer to the gas retention properties of dough samples, and the maximum height of the dough (Hm [mm]) and the total time elapsed to reach maximum dough development height (T1) are a composite reflection of gas production ability and retention capacity of the dough sample. In fresh dough samples, the presence of BaAFP-1 decreased V(CO_2_) and increased R, resulting in no significant (*p* > 0.05) difference in Hm and T1. These results were in contrast to those of the TSISPs described by Jia [34]. BaAFP-1 is homologous with alpha-amylase inhibitor BDAI-1 (H. vulgare) [21]. Though no obvious homology can be observed in the amino acid composition of AFPs purified, most of them show dual functions of AFPs and pathogenesis-related proteins which have antimicrobial activity by targeting molecules in the cell wall of bacteria or fungi [35]. Thus, the addition of BaAFP-1 may inhibit yeast activity in fresh dough samples to some extent, consequently decreasing the gas production ability of the dough. The microstructural observations described above showed that the addition of BaAFP-1 strengthened the gluten network, decreased the detachment of starch granules, consequently increasing gas retention properties. As these two effects cancelled each other, no significant difference was found in Hm or T1 in fresh dough samples. After freeze-thaw cycles, Hm and T1 decreased significantly (*p* < 0.05), indicating that freeze-thaw treatment decreased both the gas production and the retention capacities of dough samples. These effects have previously been reported by Bhattacharya [7] and Smail [36]. They attributed these effects to physical damage of the gluten network and a decrease in yeast viability and activity caused by ice crystal formation and recrystallisation. Changes in water mobility and water migration during frozen storage are also result in proofing power decrease [37]. It has been reported that BaAFP-1 weakens the deterioration of frozen dough during freeze-thaw treatment as it not only influence the freezable water content and water mobility, but also influence water distribution in dough samples [18]. No significant differences (*p* > 0.05) were observed between Control and BaAFP-1 dough samples after first freeze-thaw cycle in this study. This may be due to the inhibition of yeast activity by BaAFP-1. Moreover, BaAFP-1 would protect the gluten network and yeast from deterioration caused by formation during the freezing process [11]. Hm and T1 decreased significantly (*p* < 0.05) in both control and BaAFP-1-containing dough samples during freeze-thaw cycles. This was because large ice crystals formed due to ice recrystallisation, which occurs during the freeze-thaw cycle. These ice crystals cause mechanical damage to yeast, which reduces its survival rate and leads to a decrease in yeast viability [5]. Reducing material, such as glutathione, released by damaged yeast cells would destroy the gluten network [38]. The change in osmotic pressure in the dough system also has a major inhibitory effect on yeast activity. After four repeated freeze-thaw cycles, both the gas production and retention properties of BaAFP-1-containing dough samples were significantly (*p* < 0.05) higher than those of control. This indicated that, during freeze-thaw cycles, BaAFP-1 protected gluten network and yeast activity, thus reducing the decline rate in the overall quality of the dough due to its high ice recrystallisation inhibition activity. These results were consistent with the previously study on its effect on thermal properties and water state of dough [18] and the dynamic rheological data of the dough samples, as discussed above. 

### 3.4. Effect of BaAFP-1 on the Baking Properties of Dough Samples 

**Figure 3:**
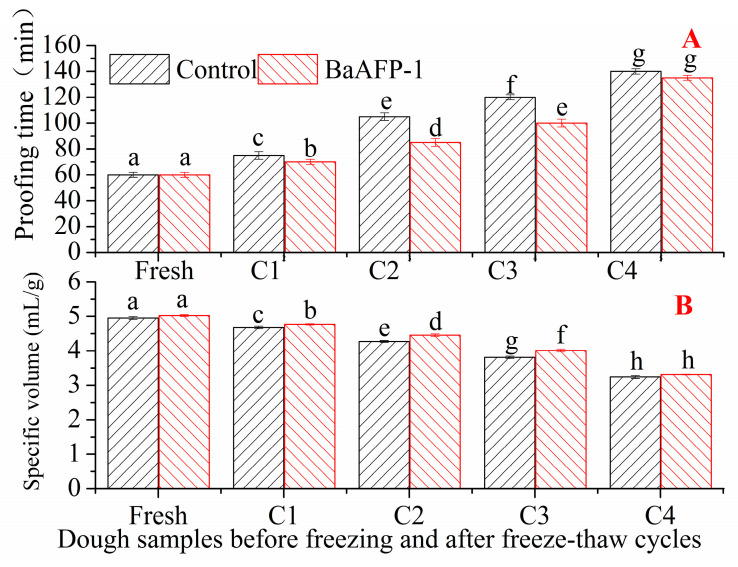
Effect of BaAFP-1 on the proofing time of dough samples (**A**) and specific volume of bread samples (**B**).

Effect of BaAFP-1 on the baking properties of dough samples including the proofing time and the specific volume are shown in Figure 3A,B, respectively. Among fresh dough samples, the proofing time was slightly shorter for control dough samples than for BaAFP-1-containing dough samples, and no significant difference (*p* > 0.05) cam be observed in the specific volume between them. After the first freeze-thaw treatment, the proofing time of both dough samples was significantly (*p* < 0.05) prolonged, accompanied by a significantly (*p* < 0.05) decrease in their specific volume. This may be because the freezing process exposes the dough to an extremely low temperature for the first time, which destroys the gluten network and decreases the number and viability of yeast cells. Due to the cryoprotective effect of BaAFP-1, the gluten network and yeast cells were protected at the presence of BaAFP-1, thus the proofing time was shorten. After a freeze-thaw cycle, the proofing time was significantly shorter and the specific volume was significantly larger for BaAFP-1-containing dough samples than for control dough samples (*p* < 0.05). This significant difference (*p* < 0.05) can be observed also after two to three freeze-thaw treatment both in the proofing time of bread dough and in the specific volume of bread. After successive freeze-thaw treatments, no significant differences (*p* > 0.05) can be observed between two dough samples. The reason for this finding may be that the cryoprotective effect of BaAFP-1 was not great enough to protect against the severe deterioration caused by multiple freeze-thaw cycles. It is worth mentioning that although significant difference in specific volume can be observed between two dough samples, their weight was similar. Because the baking pan restricted the length and width of bread samples, the difference in specific volume may be due to differences of bread height. As dough samples were controlled at unified height after proofing, the differences in bread crumb height after baking were mainly influenced by gas expansion effects and gas holding capacity of dough samples during baking. Gas expansion effect and gas holding capacity of dough samples are determined by the gas production capacity of the yeast cells and the gas holding capacity of the gluten network, which are both influenced by freezing injury during freeze-thaw cycles. On the macro level, differences were observed in the number of gas pores and the thickness of the gas cell wall between the two bread samples. Numerous small gas pores with thin gas cell walls in bread crumbs, based on a large specific volume, are required to produce high quality bread [12]. Research by Jia and Jia et al. showed that, during frozen storage, the presence of the TSISP extract from Chinese privet (*Ligustrum vulgare*) leaves decreased the yeast cell survival rate, shortened the proofing time of the bread dough, and inhibited the decrease in bread specific volume [11,34]. Xu et al. reported that ISPs from white wheat were highly effective at improving baking properties when prolonged frozen storage and freeze-thaw cycles were proceeded [39]. Similar results were obtained with BaAFP-1 here. 

### 3.5. Effect of BaAFP-1 on Bread Crumb Hardness 

**Figure 4:**
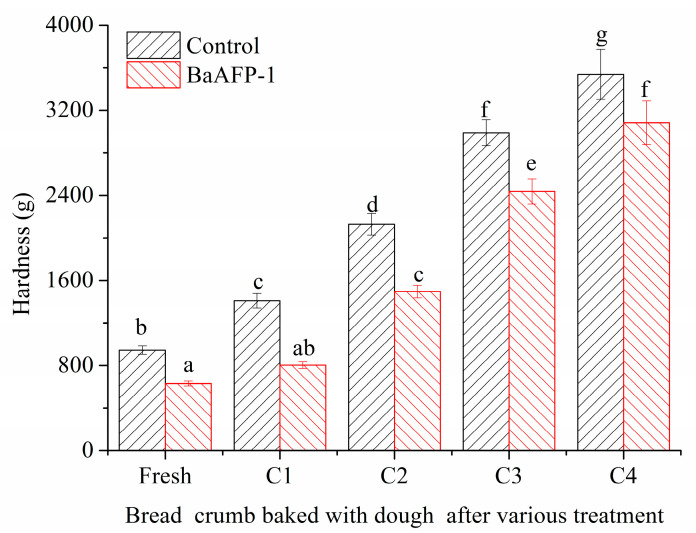
Effect of BaAFP-1 on the hardness of bread crumb.

Effect of BaAFP-1 on the hardness of bread crumb samples is shown in Figure 4. The presence of BaAFP-1 significantly (*p* < 0.05) decreased the hardness of bread crumbs baked with fresh dough samples. Similar results have previously been reported with plant AFPs and antifreeze peptides from pig skin collagen in frozen dough [40]. The addition of water-soluble polymers, such as carboxymethyl cellulose, gums, and modified starch, which have strong water absorption capacity, improve the water holding capacity of bread dough, resulting in the retention of CO_2_ during the proofing and baking processes, a larger volume, improved flavour, and decreased hardness [9,41]. Thus, the decreased hardness of bread crumbs baked with fresh dough may be influenced by the high hydrophilicity of BaAFP-1. The hardness of all bread crumbs baked with dough samples that had undergone freeze-thaw cycles increased significantly (*p* < 0.05), with a relatively slower trend in samples supplemented with BaAFP-1. The influences of freezing process and frozen storage on the integrity of hydrated gluten [1,2], the structural and functional properties of wheat starch [3], and the viability and activity of yeast [4] were reflected in the final products as prolonged proof time, reduced specific volume, and deteriorated textural characteristics. It has been reported that hardness is inversely correlated with the specific volume of bread, and thus, a lower specific volume of bread results in greater hardness due to denser crumbs and more compact cells [42]. Similar results were obtained here because a decreased specific volume was observed in dough samples after freeze-thaw cycles, as discussed above. Soy peptides and glutathione could improve baker’s yeast tolerance to freeze-thaw stress [4,43]. Their cryoprotective mechanism is thought to be the reduction in intracellular freezable water content due to their high hydrophilicity. ISPs reduce syneresis and decrease the hardness of corn and wheat starch gels by decreasing the size of ice cell cavities [44]. They are also effective in influencing the water-holding capacity and bread-making properties of frozen dough. Moreover, the water-holding capacity has a strong relation with bread-making properties of frozen dough [39]. Thus, the lower hardness increase rate in the of BaAFP-1-containing bread crumbs made from dough after undergoing multiple freeze-thaw cycles may be not only due to the unique THA and IRI of BaAFP-1, but also due to its high hydrophilicity. 

### 3.6. Effect of BaAFP-1 on the Grain Features of Bread Crumbs 

**Figure 5:**
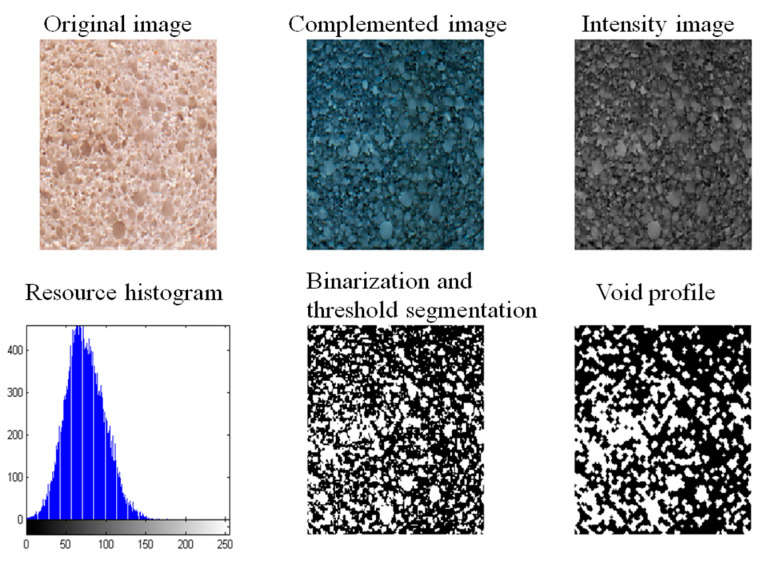
Convention, identification and analysis of the image of bread pore distribution.

The evaluation of the porous crumb structure quality of leavened baked goods, especially bread, has become a vast study area. Bread crumbs with a sufficient loaf volume and high porosity are considered to be of high quality [45]. Images of pore distribution in bread crumbs were converted and analysed with MATLAB. A typical image after conversion is present in Figure 5, and the data obtained from the images is present in Table 2. From the data presented in Table 2, it can be inferred that the average area of fresh BaAFP-1 bread crumb was significantly lower than that of control (*p* < 0.05), whereas the number of fresh BaAFP-1 bread crumb was significantly higher than that of control (*p* < 0.05). Before freezing, the viability and activity of the yeast in the dough samples was confirmed to ensure a sufficient amount of gas was produced during fermentation. The gluten network developed with good elasticity and ductility, and the starch bound to gluten helped increase the gas-holding capacity. Numerous large pores with thin pore walls were formed in the bread. When part of the pore wall was too thin to detect, it was considered to be a large continuous pore. Thus, a small number of pores with a large average area were produced. In the fermentation properties discussed above, no significant difference (*p* > 0.05) can be observed in the total volume of gas produced and significant difference (*p* < 0.05) can be observed in the retention volume of gas. Significant difference (*p* < 0.05) found in the number and the average area in fresh bread crumbs verified fermentation properties discussed above. 

During freeze-thaw treatment, the average area of bread crumb pores decreased significantly (*p* < 0.05) as the total number of pores increased significantly (*p* < 0.05). This implied that freezing and freeze-thaw treatment deteriorated the internal structure of the bread. After two to three freeze-thaw treatments, the average area of BaAFP-1 bread crumb was significantly lower than that of control (*p* < 0.05), whereas the number of BaAFP-1 bread crumb was significantly higher than that of control (*p* < 0.05). This implied that the addition of BaAFP-1 could inhibited the deterioration effect caused by freeze-thaw treatments. This result was due to the combined effects of freezing process and freeze-thaw treatment on the starch, gluten, and yeast in the dough samples. Structure of gluten and starch was destroyed by both ice crystals formation and their recrystallisation, and the viability and activity of yeast decreased. Consequently, gas formed by yeast and retained by the gluten network both decreased. Thus, the average pore area also decreased. The amount of gas retained in the dough was insufficient to extend the gluten network and thick pore walls were formed, as reflected by increased pores. The protective effect of AFPs on the gluten network, starch, and yeast in dough has previously been reported [1,11,44]. This was confirmed here, with BaAFP-1 reducing the rate of change in the number and average area of pores after freeze-thaw treatment. 

## 4. Conclusions

Here, we examined the effect of BaAFP-1 on bread and bread during freezing process and freeze-thaw cycles. Rheological properties, microstructure, fermentation, and baking performance including the proofing time and the specific volume of bread dough, textural and grain feature of bread crumbs were observed. BaAFP-1 could slow down the decrease rate of G′ and G′′of dough during freezing process and freeze-thaw treatment, and the destructive effect of freezing and freeze-thaw cycles was found to be higher on glutenins than on gliadins. This influenced the formation and the shape of ice formed in dough samples during freezing, and resulted in the inhibition of ice recrystallisation during freeze-thaw treatment. BaAFP-1 decreased the V(CO_2_) but increased the R value of fresh dough. Both the gas production and retention capacities were higher in BaAFP-1-containing dough than control dough after freeze-thaw treatment. After freezing and freeze-thaw treatment. the proofing time of both dough samples was prolonged accompanied by a decrease in their specific volume. The presence of BaAFP-1 could protect the gluten network and yeast cells, as indicated by shorter proofing times and larger specific volumes of BaAFP-1-containing dough samples. No significant difference in proofing time or specific volume were observed between the two dough samples after successive freeze-thaw treatments (*p* > 0.05). The presence of BaAFP-1 significantly (*p* < 0.05) decreased the hardness of bread crumbs baked with fresh dough samples., Bread crumb hardness increased significantly in all samples (*p* > 0.05) after freeze-thaw cycles, but at a relatively slower rate in BaAFP-1-containing samples. The average area of pores in bread crumbs decreased significantly as the total number of pores increased significantly (*p* > 0.05), and the addition of BaAFP-1 inhibited this deterioration. These results confirmed the cryoprotective activity of BaAFP-1 in bread dough during freezing and freeze-thaw cycles. Bread dough is a complex matrix. It contains several ingredients, such as gluten matrix, starches, and yeast, that can affect final properties of bread. Although the cryoprotective effect of BaAFP-1 have been proved during freezing process and freeze-thaw treatments, the actual effect of BaAFP-1 on gluten matrix, starches and yeast, respectively, can not be revealed because ingredient interacted with each other. More efforts should be put on the cryoprotective effect of AFPs on gluten matrix, starches, and yeast via artificial dough systems which can identify the interaction of these structural elements in more detail.

## Figures and Tables

**Table 1:** Effect of BaAFP-1 on fermentation properties of frozen dough after freeze-thaw treatment.

		Hm	T1	Hm’	Total Volume	Retention Volume	R
Control	Fresh	33.2 ± 1.13 ^a^	57 ± 1.41 ^a^	52.7 ± 0.42 ^a^	1233 ± 2.83 ^a^	1111 ± 0.71 ^a^	90.1 ± 0.28 ^a^
	C1	27.1 ± 0.28 ^b^	73 ± 0.71 ^b^	45.1 ± 1.27 ^c^	1078 ± 2.83 ^c^	992 ± 2.12 ^c^	92.0 ± 0.71 ^c^
	C2	13.1 ± 0.28 ^e^	107 ± 0.00 ^e^	38.4 ± 0.57 ^d^	930 ± 1.41 ^e^	846 ± 4.95 ^f^	90.9 ± 0.28 ^b^
	C3	12.5 ± 0.35 ^e^	125 ± 1.41 ^f^	30.5 ± 0.78 ^f^	751 ± 0.00 ^g^	717 ± 0.71 ^i^	95.4 ± 0.28 ^ef^
	C4	7.0 ± 0.14 ^g^	180 ± 2.12 ^h^	29.1 ± 0.28 ^f^	721 ± 1.41 ^h^	692 ± 0.71 ^j^	95.9 ± 0.42 ^f^
BaAFP-1	Fresh	34.2 ± 0.21 ^a^	58 ± 0.71 ^a^	53.2 ± 1.41 ^a^	1148 ± 3.54 ^b^	1092 ± 3.54 ^b^	95.1 ± 0.71 ^e^
	C1	28.1 ± 0.07 ^b^	70 ± 2.83 ^b^	47.4 ± 0.92 ^b^	1004 ± 0.00 ^d^	959 ± 0.71 ^d^	95.4 ± 0.28 ^ef^
	C2	20.2 ± 0.21 ^c^	86 ± 2.12 ^c^	39.0 ± 1.41 ^d^	934 ± 0.71 ^e^	867 ± 0.71 ^e^	92.8 ± 0.71 ^d^
	C3	15.3 ± 0.42 ^d^	102 ± 2.12 ^d^	35.7 ± 0.21 ^e^	817 ± 1.41 ^f^	735 ± 2.12 ^g^	89.9 ± 0.42 ^a^
	C4	11.2 ± 0.07 ^f^	136 ± 2.12 ^g^	29.6 ± 0.28 ^f^	752 ± 0.71 ^g^	729 ± 0.71 ^h^	96.9 ± 0.42 ^g^

Mean value ± SD with different superscript letters (a, b, c, d, e, f, g, h, i, j) in the same column are significantly different (*p* < 0.05).

**Table 2:** Number and average area of bread pore obtained by image analysis.

		Number	Average Area (mm^2^)
Control	Fresh	508.46 ± 8.32 ^b^	125 ± 0.71 ^b^
	C1	551.15 ± 8.70 ^c^	113 ± 1.41 ^c^
	C2	699.42 ± 12.03 ^d^	106 ± 2.12 ^d^
	C3	881.46 ± 7.70 ^f^	84 ± 1.41 ^f^
	C4	1232.10 ± 7.94 ^h^	60 ± 1.41 ^h^
BaAFP-1	Fresh	469.17 ± 12.99 ^a^	134 ± 2.83 ^a^
	C1	567.87 ± 14.62 ^c^	112 ± 2.83 ^c^
	C2	691.60 ± 5.97 ^d^	93 ± 2.12 ^e^
	C3	809.66 ± 8.22 ^e^	90 ± 1.41 ^e^
	C4	989.93 ± 3.10 ^g^	78 ± 2.12 ^g^

Mean value ± SD with different superscript letters (a, b, c, d, e, f, g, h) in the same column are significantly different (*p* < 0.05).

## Supplementary Materials

The following are available online at https://www.mdpi.com/2304-8158/9/11/1698/s1, Program S1: MATLAB program used in the grain features determination of bread crumbs.
