# Supplementary material for: Effect of Barley Antifreeze Protein on Dough and Bread during Freezing and Freeze-Thaw Cycles
Source: Foods. 2020 Nov 19;9(11):1698. doi: 10.3390/foods9111698 (PMC7699476; doi:10.3390/foods9111698)
Supplement: Supplementary file 1 [file foods-09-01698-s001.pdf]

MATLAB program used in the grain features determination of bread crumbs

```
[I,map]=imread('C:\Users\Administrator\Desktop\name of image');
hold on;subplot(231);imshow (I,map);title('Original image');
I1 =imcomplement (I);% invert
hold on;subplot(232);imshow (I1);title('Complemented image');
I2=rgb2gray(I1);
hold on;subplot(233);imshow(I2); title('Intensity image');
hold on;subplot(234); imhist(I2);title('Resource histogram');
axis([0 200 0 500]);
level = graythresh(I2);
imgbw = im2bw(I2,level);
hold on;subplot(235);imshow(imgbw);title(' Binarization and threshold segmentation ');
se=strel ('disk', 1);% Expansion determines the structure of the element
I3=imclose (imgbw,se);% form close
I4=imopen(I3,se);% form open
hold on;subplot(236);imshow(I4);title('Void profile ');
I5=I3&I4;
 [r,num]=bwlabel(I5,4);
L=regionprops(r, 'area');
area=cat(1,L. Area);
totalarea=sum(area);
ratio=totalarea/(382*382);
averagearea=sum(area)/num;
```
